# Ectopic burst induced by blockade of axonal potassium channels on the mouse hippocampal mossy fiber

**DOI:** 10.3389/fncel.2024.1434165

**Published:** 2024-07-04

**Authors:** Haruyuki Kamiya

**Affiliations:** Department of Neurobiology, Hokkaido University Graduate School of Medicine, Sapporo, Japan

**Keywords:** axon, burst firing, ectopic spike, hippocampus, mossy fiber

## Abstract

A potassium channel blocker 4-AP has been shown to exert pronounced convulsive action to generate burst firings when applied to hippocampal slices. However, it remains unclear how the blockade of potassium channels leads to the generation of burst firings. One possibility is ectopic spiking from the sites different from those for physiological spike initiation at the axon initial segment, as suggested for several experimental models of epileptogenesis *in vitro*. To test for possible ectopic spiking at the distal axon by 4-AP application, direct recordings from large mossy fiber terminals were made with the loose-patch clamp technique in mouse hippocampal slices. To localize the action of 4-AP on the distal axon, focal perfusion, as well as micro-cut to disconnect soma and distal axons, were adopted. Focal application of 4-AP on the distal portion of mossy fibers reliably induced burst discharges of the mossy fiber terminals. Photochemical blockade of potassium channels at distal axons, by the application of RuBi-4-AP, a visible wavelength blue light-sensitive caged compound, and the illumination of blue light caused robust bursting activity originating from distal axons. Computer simulation suggested that local blockade of axonal potassium channels prolongs the duration of action potentials and thereby causes reverberating spiking activities at distal axons and subsequent antidromic propagation toward the soma. Taken together, it was suggested that local blockade of voltage-dependent potassium channels in distal axons by application of 4-AP is sufficient to cause a hyperexcitable state of hippocampal mossy fiber axons.

## Introduction

Epilepsy is one of the common neuronal disorders, characteristic of causing the hyperexcitability of neuronal networks in the central nervous system (Gutnick and Prince, 1972; Netoff and Schiff, 2002; Salami et al., 2016). To understand the cellular and network mechanisms underlying epilepsy, several experimental conditions have been reported to cause a hyperexcitable state of neuronal networks *in vitro*. These include suppression of GABAergic inhibition by application of picrotoxin or bicuculline (Chesnut and Swann, 1989), enhanced glutamatergic excitation by application of kainic acid (Fisher and Alger, 1984), and inhibition of K^+^ channels by 4-AP (Avoli et al., 1988; Chesnut and Swann, 1988, 1990; Brückner et al., 1999; Carriero et al., 2010; Lévesque et al., 2013). Among these, detailed mechanisms of 4-AP-induced epileptogenesis remained to be elucidated (Siniscalchi and Avoli, 1992; Yamaguchi and Rogawski, 1992; Mattia et al., 1994; Kapetanovic et al., 1995). The primary action of 4-AP is blocking a certain class of voltage-gated K^+^ channels (Buckle and Haas, 1982; Storm, 1988; Perreault and Avoli, 1989; Rama et al., 2017). The application of 4-AP has been shown to slow down repolarization and thereby prolong the duration of action potentials propagating along the hippocampal mossy fiber axon (Geiger and Jonas, 2000; Alle et al., 2011). However, it remains to be determined how prolonged axonal action potentials lead to cellular and network oscillations in hippocampal neuronal networks. Axonal hyperexcitability was demonstrated to accompany several models of epileptogenesis *in vitro* such as a stimulus-induced burst of axons by repetitive stimulus trains (Stasheff et al., 1993) as well as a kainate-induced oscillation of ɣ-range activities (Dugladze et al., 2012). To test for the possible contribution of axonal hyperexcitability in 4-AP-induced epileptogenesis in the hippocampal CA3 region, it was attempted to localize the 4-AP action in a combination of direct recordings from the single mossy fiber axon terminals (Ohura and Kamiya, 2018a), as well as focal perfusion, uncaging, and physical disconnection with a micro-cut. All experimental evidence supports the notion that the burst discharges originate ectopically from the distal portions of the axons. The computational simulation also supports this notion by reconstructing burst discharges by removing potassium conductance from distal axons. Taken together, it was suggested that distal axons may serve as a cellular oscillator for reverberating burst discharges under the condition of blockade of potassium conductance.

## Materials and methods

### Ethical approval

All animals were treated according to the guidelines for the care and use of laboratory animals at Hokkaido University. All procedures were approved by the local committee at Hokkaido University (#23–0040).

### Preparation of hippocampal slices

C57BL/6J mice were initially purchased (Japan SLC, Hamamatsu, Japan) and later bred in-house. Transverse hippocampal slices 300 μm thick were prepared from C57BL/6J mice of either sex (p16-p51, number of animals = 40) as described previously (Shimizu et al., 2008) with some modifications. Animals were anesthetized with isoflurane and the brain was dissected in an ice-cold sucrose solution containing the following (in mM): 40 NaCl, 25 NaHCO3, 10 glucose, 150 sucrose, 4 KCl, 1.25 NaH_2_PO_4_, 0.5 CaCl_2_, and 7 MgSO_4_ (Geiger et al., 2002). Transverse hippocampal slices were cut using a VT1200S microslicer (Leica Biosystems, Germany), and the above solution was replaced with an NMDG-HEPES recovery solution containing the following (in mM): 93 NMDG, 30 NaHCO_3_, 25 glucose, 20 HEPES, 2.5 KCl, 1.2 NaH2PO_4_, 5 Na-ascorbate, 2 Thiourea, 3 Na-pyruvate, 0.5 CaCl_2_, 10 MgSO_4_, and incubated for no longer than 15 min (Ting et al., 2014). Then, the solution was exchanged again with artificial cerebrospinal fluid (ACSF) containing the following (in mM): 127 NaCl, 1.5 KCl, 1.2 KH_2_PO_4_, 26 NaHCO_3_, 10 glucose, 2.4 CaCl_2_, and 1.3 MgSO_4_, and the slices were kept in an interface-type chamber saturated with 95% O_2_ and 5% CO_2_ at room temperature (around 25°C).

### Electrophysiology

**Figure 1:**
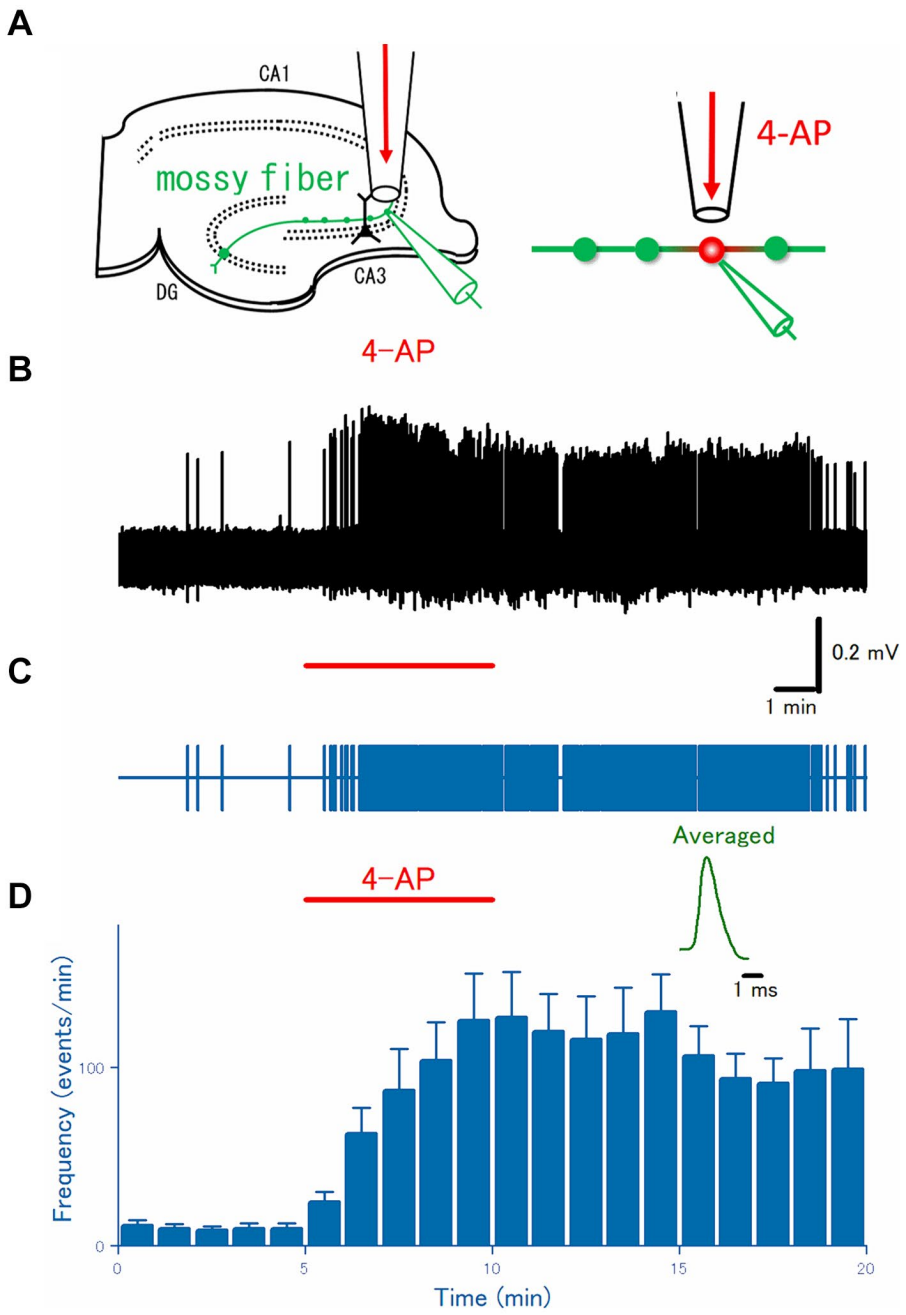
Axonal burst discharges by focal application of 4-AP to the distal portions of the hippocampal mossy fibers. **(A)** Schematic drawing of the experimental arrangement. Loose-patch recordings were obtained from visually-identified mossy fiber boutons. The surrounding region of the recording site was focally perfused with a continuous flow of perfusate through a flow pipe and switched to that containing 100 μM 4-AP for 5 min. **(B)** A representative trace of the axonal spikes was recorded from the single mossy fiber bouton for 20 min. **(C)** A raster plot of data is shown in **(B)**. **(D)** Time course of the frequency of the axonal spikes during 4-AP application as shown by the red bar (*n* = 15). Inset represents the averaged waveforms of axonal spikes in **(B)**.

The slices were perfused with the ACSF at around 2 mL/min and maintained at 24–26°C in a recording chamber. In addition, the slice surface of the recording site was locally perfused with the standard or Ca^2+^-free ACSF (equal concentration of Mg^2+^ was replaced for Ca^2+^; 0 CaCl_2_ and 3.7 MgSO_4_) at about 0.2 mL/min through a flow pipe with a 250 μm open-tip diameter connected to an electromagnetic valve system (Valve Bank; Automate Scientific, Berkeley, CA, United States) for faster exchange of solution selectively around the recording sites (Figure 1A), as described previously (Kamiya, 2012). The Ca^2+^-free ACSF was used to suppress all synaptic transmission and therefore eliminate possible recording from postsynaptic neurons.

For the extracellular recording of axonal spikes from single mossy fiber boutons, glass pipettes containing the extracellular solution (typically 3–6 MΩ electrode resistance) were placed on the visually identified putative boutons in the stratum lucidum under an IR-DIC microscope (BX51WI, Olympus, Tokyo, Japan), and gentle suction was applied to the recording pipettes. A loose patch configuration was used to achieve less invasive stable recording from the small boutons (Ohura and Kamiya, 2018a). For instance, even under continuous focal perfusion around the recoding site (see above, Figure 1A), stable recordings for long periods are readily feasible and therefore are suited for the quantitative pharmacological study of the bath or focally applied drugs.

All recordings were made at room temperature (24–26°C). Extracellular axonal spikes were recorded with glass pipettes using a Multiclamp 700B amplifier (Molecular Devices, San Jose, CA, United States). Signals were filtered at 10 kHz with a 4-pole Bessel filter, sampled at 20 kHz, and analyzed offline with pCLAMP10 software (Molecular Devices, San Jose, CA, United States).

### Simulation

The simulated membrane potential (*V*_m_) at the hippocampal mossy fibers was calculated according to the model suggested by Engel and Jonas (2005) based on the data recorded from mossy fiber boutons. The simple mossy fiber model was reconstructed by implementing the structure of *en passant* axons as well as experimentally obtained properties of ionic conductances (Engel and Jonas, 2005) and was uploaded to the ModelDB database as accession no. 263034.^1^ The model assumed a Hodgkin-Huxley-type model adapted to channels in mossy fiber terminals, and K^+^ channel inactivation was implemented multiplicatively with parameters of recombinant K_V_1.4 channels (Wissmann et al., 2003). Simulations were performed using NEURON 8.2 for Windows (Hines and Carnevale, 1997). The passive electrical properties of the axon were assumed to be uniform, with a specific membrane capacitance *C*_m_ of 1 μF cm^−2^, a specific membrane resistance *R*_m_ of 10,000 Ω cm^2^, and an intracellular resistivity *R*_i_ of 110 Ω cm. The structure of the mossy fiber was approximated by a soma (diameter, 10 μm), 10 axonal cylinders (diameter, 0.2 μm; length, 100 μm), and 10 *en passant* boutons (diameter, 4 μm). The number of segments was 1 μm^−1^, and the time step was 5 μs in all simulations. The resting potential was assumed to be −80 mV, and the reversal potential of the leak conductance was set to −80 mV. Voltage-gated Na^+^ channels, K^+^ channels, and leakage channels were inserted into the soma, axon, and boutons, respectively. The Na^+^ conductance density was set to 50 mS cm^−2^ for the axons and boutons and 10 mS cm^−2^ for the soma. The K^+^ conductance density was set to 36 mS cm^−2^ throughout all parts of the neurons. Action potentials were evoked by the injection of a depolarizing current into the soma (2 ms, 0.2 nA). *V*_m_ at the last (10th) bouton in a “pearl chain structure” was calculated in the simulation. The equilibrium potentials for Na^+^ and K^+^ ions were assumed to be +50 mV and −85 mV, respectively. All model and simulation files will be available from the ModelDB database as accession no. 2015571^2^.

### Statistics

All data are expressed as the mean ± SEM, and *n* represents the number of recording boutons. Statistical analysis was performed by non-parametric two-sided tests (Wilcoxon signed-rank test for paired data and Mann–Whitney *U* test for unpaired data), and a *p*-value of <0.05 was accepted for statistical significance.

### Chemicals

All chemicals were purchased from Wako Pure Chemical Industries (Tokyo, Japan), except for RuBi-4-AP purchased from Tocris (Bristol, United Kingdom).

## Results

### Focal application of 4-AP induced ectopic burst at single mossy fiber boutons

To directly test for the generation of burst discharges from distal axons during the application of 4-AP, axonal spikes were recorded from the single mossy fiber boutons visually identified under IR-DICs optics with the aid of a high NA (1.0) objective (Olympus, Tokyo, Japan). As reported previously (Ohura and Kamiya, 2018a), given the characteristic size of 4–7 μm in diameter, localization at stratum lucidum, and all-or-none responsiveness to the stimulus given to the granule cells above the threshold intensity, it is readily possible to establish loose-patch recordings of axonal spikes from the single mossy fiber boutons. After checking all these criteria, the spontaneous axonal spikes were monitored under a focal and constant flow of normal ACSF via the flow pipe (Figure 1A). During the control period, mossy fiber boutons rarely generate spontaneous firings, as shown in Figures 1B,C. When the focal perfusate was switched to that containing 100 μM 4-AP for 5 min, a barrage of spontaneous firings was induced rapidly in the mossy fiber boutons and this effect outlasts the period of 4-AP application at least for 10 min. On average (*n* = 20), the frequency of the recorded spikes from the single mossy terminals was increased from 0.17 ± 0.050 Hz in control to 2.1 ± 0.44 Hz at 5 min after 4-AP application (*p* = 0.00005) and 1.7 ± 0.47 Hz at 10 min after washout (*p* = 0.00004), respectively (Figure 1D).

### Ectopic burst induced by 4-AP in Ca^2+^ free solution

**Figure 2:**
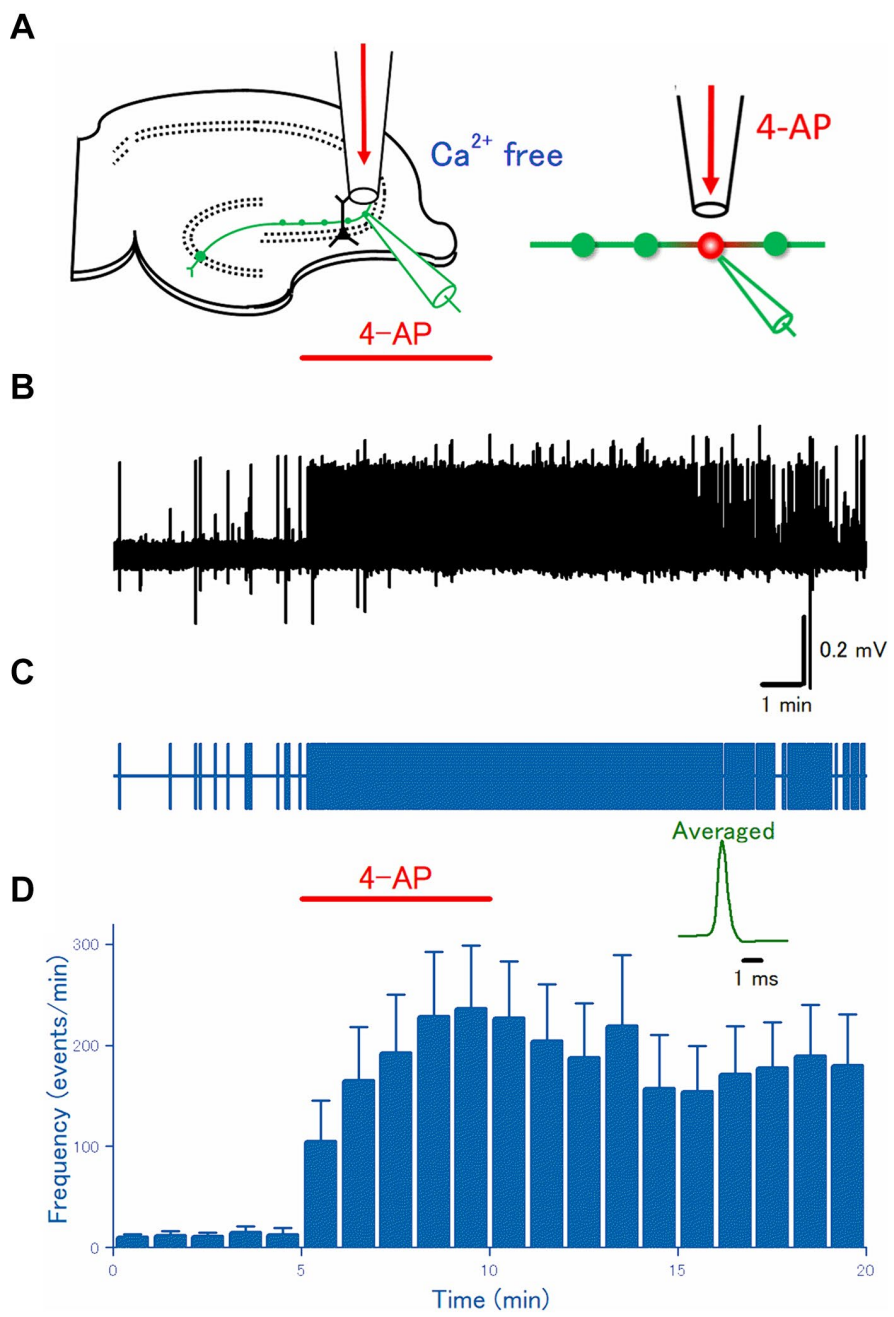
Axonal discharges elicited by 4-AP application in Ca^2+^-free solution. **(A)** An experimental configuration similar to Figure 1 was adopted, except that Ca^2+^-free ACSF was used for perfusate. **(B)** A representative trace of the axonal spikes was recorded from the single mossy fiber bouton. **(C)** A raster plot of data is shown in **(B)**. **(D)** Time course of the frequency of the axonal spikes (*n* = 24). Inset represents the averaged waveforms of axonal spikes in **(B)**.

The hyperexcitability of mossy fibers by application of 4-AP might be attributed to a direct effect on the axons or an indirect effect by changes in the surrounding microenvironment. To test whether 4-AP has direct effects on axonal excitability, first, the effect of 4-AP was examined in the Ca^2+^-free solution where the indirect action due to massively released neurotransmitters during burst activity was expected to be suppressed. Again, focal application (Figure 2A) of 100 μM 4-AP for 5 min evoked a barrage of spontaneous firings was induced rapidly in the mossy fiber boutons and this effect robustly outlasted the period of 4-AP application at least for 10 min (Figures 2B,C). On average (*n* = 25), the frequency of the recorded spikes from the single mossy terminals was increased from 0.22 ± 0.11 Hz in control to 3.9 ± 1.0 Hz at 5 min after 4-AP application (*p* = 0.00001) and 3.0 ± 0.86 Hz at 10 min after washout (*p* = 0.00001), respectively (Figure 2D). These results are in support of the idea that 4-AP-induced hyperexcitability is caused by the alteration in the cell-intrinsic property (Avoli et al., 1996) of mossy fiber axons.

It is worth noting that the frequencies of spontaneous firings before (0.22 ± 0.11 Hz) and 5 min after 4-AP application (3.9 ± 1.0 Hz) in the Ca^2+^-free condition were not different from those in the Ca^2+^-containing condition (0.17 ± 0.050 Hz and 2.1 ± 0.44 Hz, respectively) as shown in Figure 1 (*p* = 0.69 and *p* = 0.11, respectively). These findings are of significance in that the replacement of Ca^2+^ ions with identical concentrations of Mg^2+^ ions used in this study as a Ca^2+^-free condition may not modify the neuronal excitability significantly. Furthermore, it is not likely that the 4-AP-induced bursts are caused solely by the released neurotransmitters during burst activity.

### Disconnection of soma abolished 4-AP-induced burst

**Figure 3:**
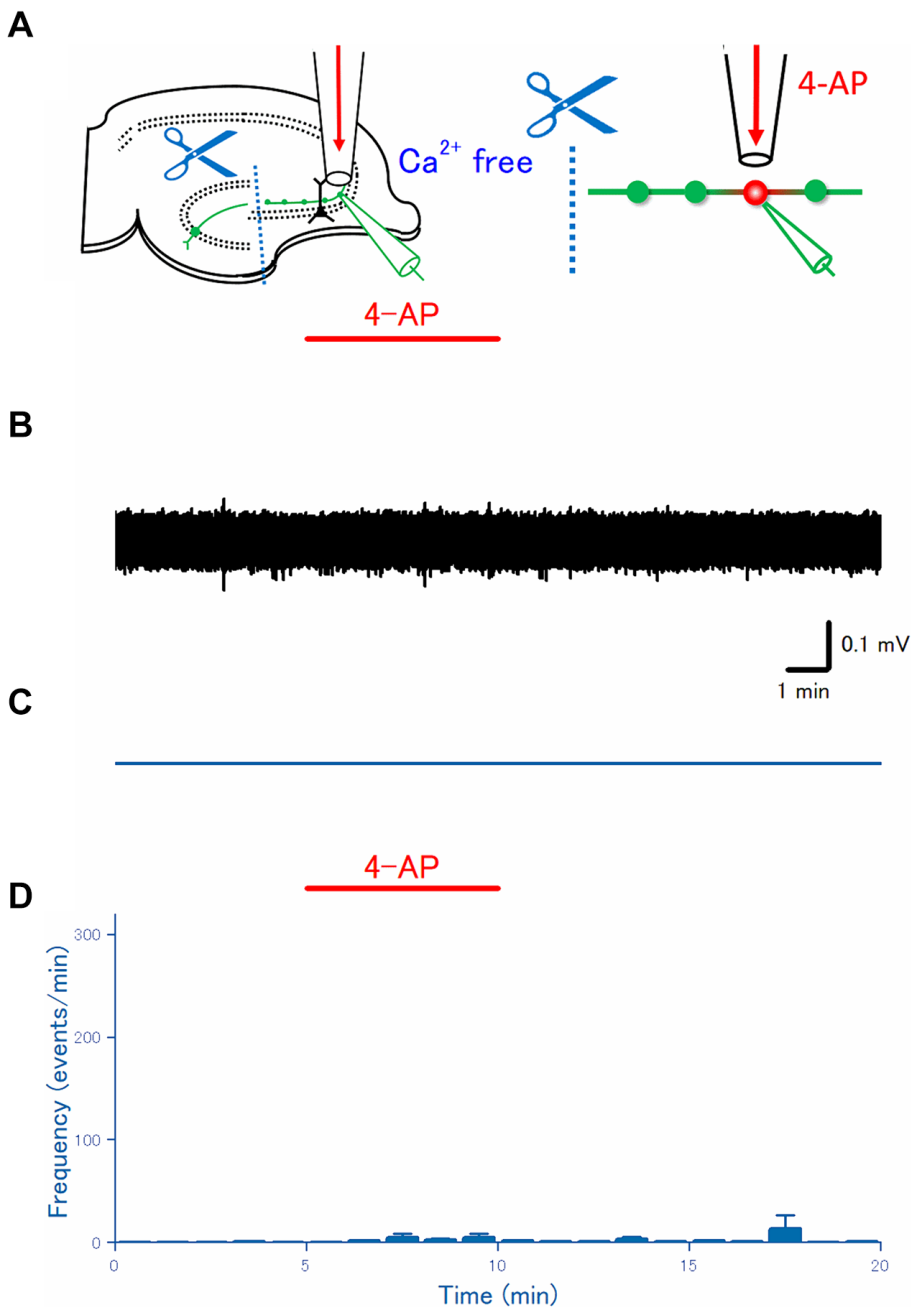
Effect of disconnecting distal axon from the soma. **(A)** The dentate gyrus was surgically cut from the CA3 region, as illustrated in the schema. **(B)** A representative trace recorded from the mossy fiber bouton. **(C)** A raster plot of data is shown in **(B)**. **(D)** Time course of the frequency of the axonal spike (*n* = 18).

To check for the ectopic origin of the 4-AP-induced burst from the distal axon, next, it was attempted to disconnect soma from the distal portion of the mossy fiber axon. Thanks to the well-organized network structure of hippocampal slices (Acsády et al., 1998; Henze et al., 2000), a micro-cut between the dentate gyrus and CA3 region was made with a surgical knife under a stereoscopic microscope, to separate the soma and the distal portion of mossy fibers (Figure 3A). As expected from the disconnection from the soma or the proximal axon, a physiological initiation site of action potentials (Schmidt-Hieber et al., 2008), action potentials were rarely recorded from mossy fiber boutons on distal axons (Figures 3B,C). On average (*n* = 18), the frequency of the recorded spikes from the single mossy terminals was 0.0028 ± 0.0020 Hz in control, 0.087 ± 0.060 Hz at 5 min after 4-AP application, 0.011 ± 0.0065 Hz at 10 min after washout, respectively (Figure 3D). The mean frequency of spontaneous spikes during the control period in the experiment with disconnection of soma (0.0028 ± 0.0020 Hz, Figure 3D) was fewer than in the experiment with intact slices (0.22 ± 0.11 Hz, Figure 2D), suggesting that some spontaneous firings of the soma are observed during the baseline recordings from the intact slices.

### Photochemical application of 4-AP by photolysis of the caged compound

**Figure 4:**
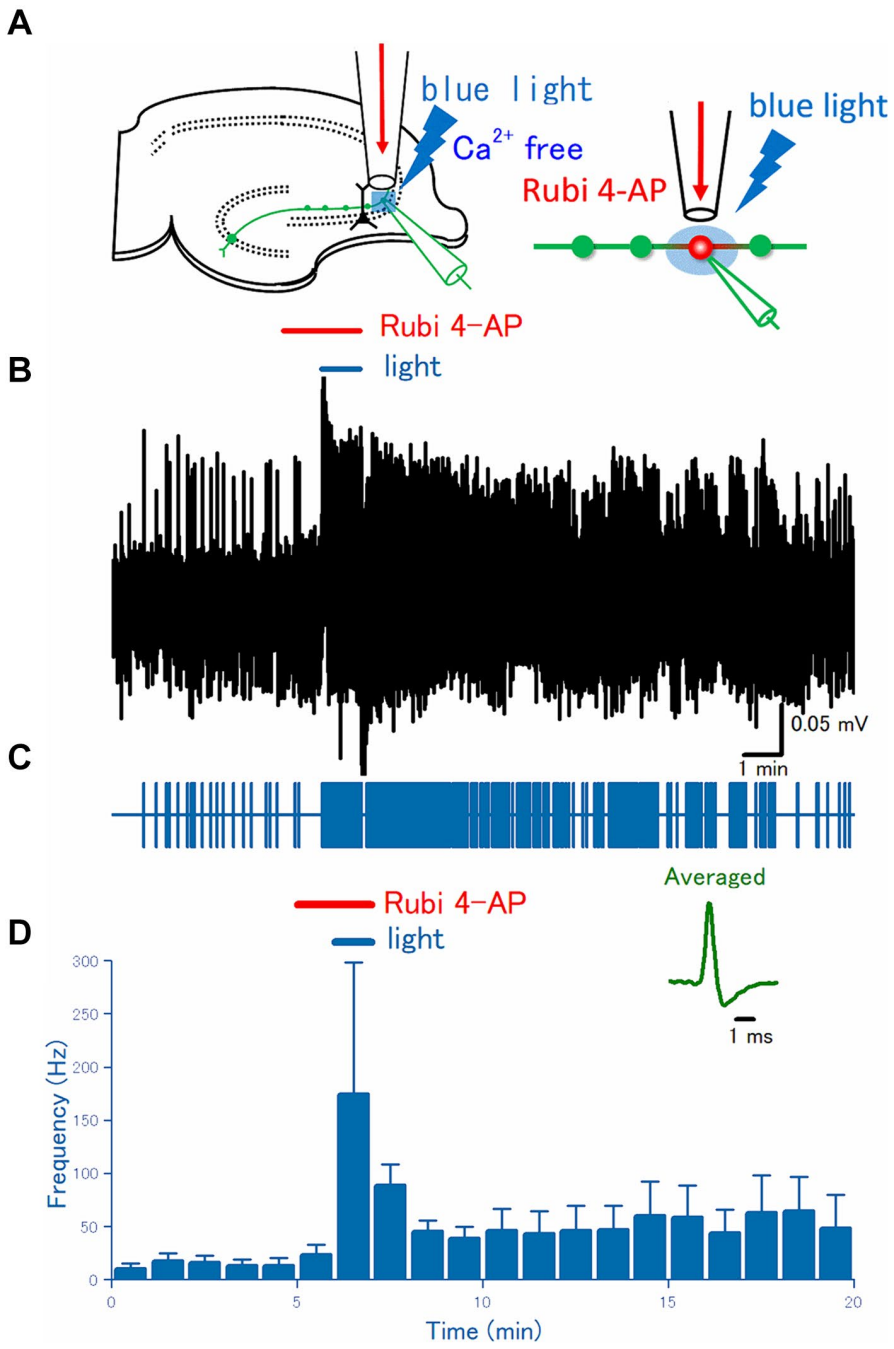
Effect of uncaging of RuBi-4-AP on the axonal discharges. **(A)** A caged compound RuBi-4-AP at 100 μM was focally applied to the recording site for 2 min, and the surrounding area was illuminated with blue light (480 nm) for the latter 1 min during RuBi-4-AP perfusion. **(B)** A representative trace of the axonal spikes recorded from the single mossy fiber bouton. **(C)** A raster plot of data is shown in **(B)**. **(D)** Time course of the frequency of the axonal spikes (*n* = 11). Inset represents the averaged waveforms of axonal spikes in **(B)**.

In the preceding experiments, 4-AP was applied through the continuous flow of the perfusate via a flow pipe, to the surface of the slices at the stratum lucidum of the CA3 region where a distal portion of mossy fiber exists. To further limit the site of 4-AP action, a photochemical approach in combination with local illumination was adopted. For this purpose, the photochemical approach using photolysis of RuBi-4-AP (Nikolenko et al., 2005) was used in this study. RuBi-4-AP is a visible wavelength blue light-sensitive caged compound based on ruthenium photochemistry and releases 4-AP upon blue light illumination (Zhao et al., 2015). In combination with local perfusion of 100 μM RuBi-4AP and illumination of 100 μm square area covering stratum lucidum of the CA3 region with blue light of 480 nm wavelength (Figure 4A) triggered burst firing promptly at the recorded single axon terminals in the illumination area (Figures 4B,C). On average (*n* = 12), the frequency of the recorded spikes from the single mossy terminals was 0.23 ± 0.12 Hz in control, 0.39 ± 0.16 Hz at 1 min after RuBi-4-AP application, 2.9 ± 2.1 Hz after 1 min blue light illumination with RuBi-4AP application (*p* = 0.00714), 0.74 ± 0.36 Hz at 10 min after washout, respectively (Figure 4D).

### Simulation of local blockade of K^+^ channels on the distal axons

All the experimental results so far supported the notion that local blockade of the axonal K^+^ channels on the distal axons is sufficient for generating the burst firing of mossy fiber axons. To convince and validate this notion quantitatively, it was attempted to perform the computer simulation using the simple model of mossy fiber axons implemented with ionic conductance determined by the direct recording experiments (Engel and Jonas, 2005).

**Figure 5:**
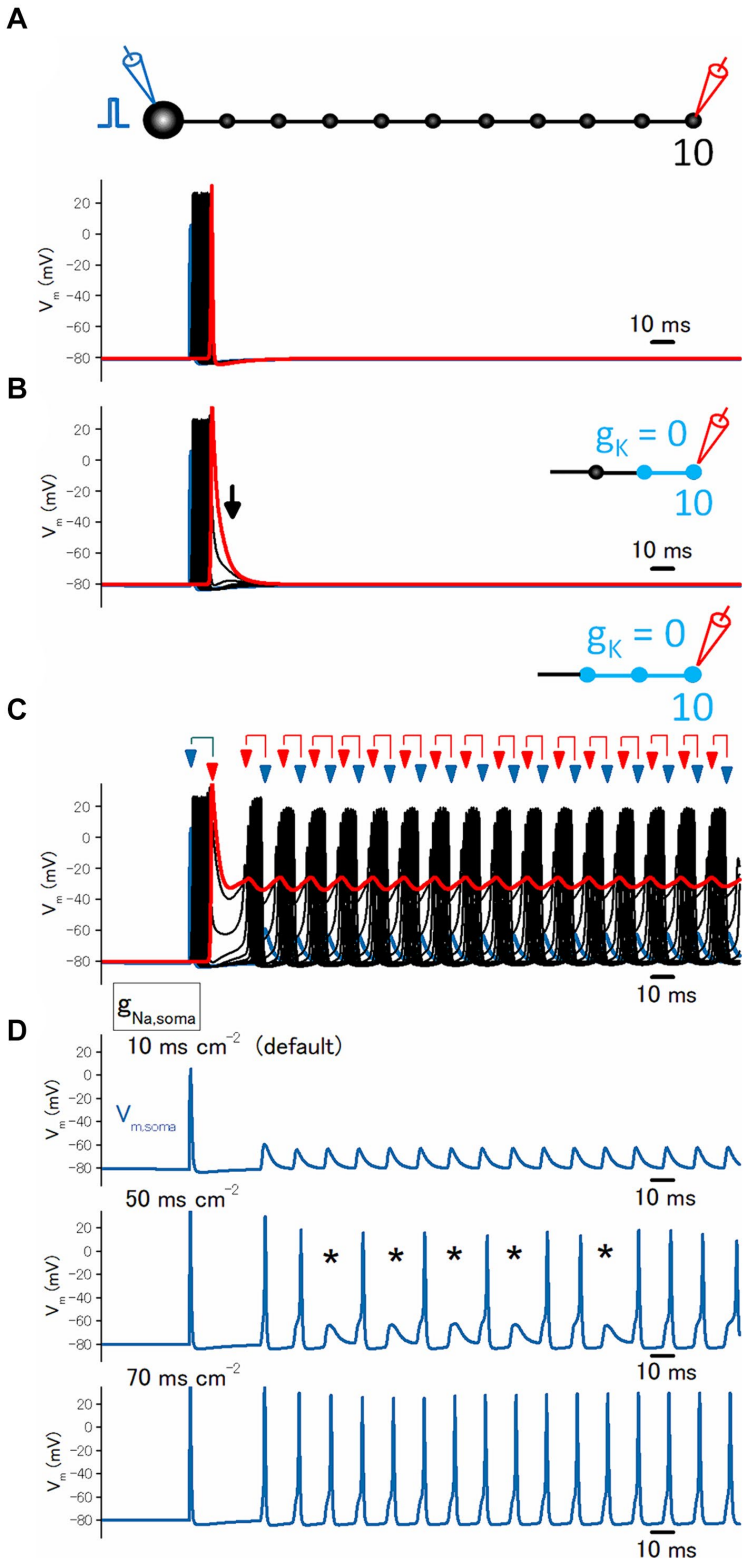
Simulation of local blockade of K^+^ channels on the distal axons. **(A)** Reliable propagation of action potential throughout en passant axon with 10 boutons evenly spaced every 100 μm (“a pearl chain model”). Brief current injection into the soma elicited action potential which propagates faithfully to the 10th bouton without attenuation. **(B)** Blocking the potassium conductance (*g*_K_) at the 9th and 10th boutons and the connecting axon shafts slowed repolarization as shown by the arrows. **(C)** Blocking *g*_K_ at the 8th to the 10th boutons and the connecting axon shafts much slowed down the repolarization phase and triggered reverberating burst discharges from the distal portion of the axons propagating antidromically toward the soma. **(D)** Traces showing the calculated membrane potentials at the soma. During the reverberating burst discharges shown in **(C)** generate spikelets at the soma (top). Increasing somatic sodium conductance (*g*_N_a) to 50 ms cm^−2^ allowed the invasion of antidromically propagated spikes in some, leaving some spikelets as shown by the asterisks (middle). Further increase in *g*_Na_ to 70 ms cm^−2^ elicited spikes in response to all reverberating activity (bottom).

In this model, stimulation of the soma with a single shock elicited axonal action potentials propagating faithfully along the axon (Figure 5A). When the potassium conductance (*g*_K_) was removed from the distal axons, the action potential at the boutons without *g*_K_ prolonged the duration of action potentials by slowing the repolarization phase (Figure 5B). By extending the regions removing *g*_K_ and once it exceeds a threshold (2 boutons in this case), the repetitive firings are triggered after the initial propagating spike (Figure 5C). It was notable that action potentials at the proximal axonal boutons occurred later than those of action potentials at the distal axonal boutons, suggesting the possibility that the repetitive firings following the initial action potential were triggered ectopically from the distal axons and propagated antidromically toward the soma. In addition, antidromic propagation is also implied by the findings that the somatic responses are small spikelets and do not generate “full” action potentials (Figure 5D, upper trace). It was speculated that antidromic invasion of axonal action potentials elicited spikelets due to impedance mismatch between thin axon shafts and large soma, and shortage of sodium conductance (*g*_Na_) required for the generation of somatic action potentials by the given antidromic spikes. In support of this notion, increasing the gNa value from the original 10^–^50 ms cm^−2^ caused action potentials and the occasional appearance of spikelets (Figure 5D, middle trace). When *g*_Na_ was increased to 70 ms cm^−2^, all axonal action potentials reliably elicited repetitive action potentials at the soma (Figure 5D, lower trace). All the simulation results support the notion that the blockade of axonal K^+^ channels leads to ectopic burst firings originating from the distal axons.

## Discussion

In this study, the mechanisms underlying hyperexcitability caused by the application of 4-AP, a blocker of potassium channels, to the hippocampal slice preparation were explored both experimentally with direct recording from the axon terminals and mathematically with computer simulation. Direct subcellular recordings from single axon terminals of the hippocampal mossy fibers, in combination with local perfusion with the flow pipe placed in the vicinity of the recording site, revealed that the ectopic burst firings originated from the distal axons by local application of 4-AP. Numerical simulation using hippocampal mossy fiber models also confirmed the notions obtained by the experiments. All the results support the ectopic burst generation by the blockade of axonal K^+^ channels on the hippocampal mossy fibers and the following antidromic propagation toward the soma.

### Hyperexcitability caused by blockade of axonal K^+^ channels

It was demonstrated that the voltage-dependent K^+^ channels shape the repolarization phase of action potentials propagating along hippocampal mossy fiber axons and determine the time course and duration (Geiger and Jonas, 2000). Application of K^+^ channel blocker 4-AP to the hippocampal slice preparation induces a broadening of action potentials (Alle et al., 2011; Carta et al., 2014) and a hyperexcitable state accompanying epileptiform afterdischarge (Chesnut and Swann, 1988), although it needs to be clarified how the broadening of action potentials by 4-AP application leads to the hyperexcitable state. In this study, it was found that the local application of 4-AP directly to the stratum lucidum caused burst firings of the recorded mossy fiber boutons. Since the 4-AP-induced burst was also induced in Ca^2+^-free ACSF, the burst firings seem not to be initiated by network activity requiring synaptic transmission but occurred by alteration of intrinsic properties of mossy fiber axons at least in part (Amir et al., 2005).

In this study, the roles of synaptic and/or glia-transmission in the 4-AP-induced bursts were tested by the replacement of Ca^2+^ ions with identical concentrations of Mg^2+^ ions to reduce the transmitter release (Figure 2). Alternatively, it may be tested to see the effect of pharmacological blockade of glutamatergic and GABAergic transmission in the physiological ionic concentrations. The pharmacological blockade is advantageous in the preserved ionic concentrations surrounding the neurons, although total block of ionotropic as well as metabotropic glutamatergic/GABAergic actions with a mixture of high concentrations of antagonists are hardly achieved practically. Transmitters different from glutamate and GABA, such as ATP (Armstrong et al., 2002) whose P2X7 type receptors exist on the mossy fiber boutons, are not blocked even in this condition. Vesicular release of zinc from the mossy fiber boutons (Wenzel et al., 1997) is also a matter of concern. These released substances such as ATP or zinc are also expected to be suppressed in Ca^2+^-free conditions. Importantly, there were no differences in the frequencies of spontaneous firings before and after 4-AP application in the Ca^2+^-free condition and in the Ca^2+^-containing condition, as illustrated in Figures 1, 2, supported the 4-AP-induced bursts are dominantly mediated by alteration of intrinsic excitability rather than the actions of neuro- and/or glia-transmitters released during the burst firings.

### Ectopic origin of afterdischarges from distal axon

It was notable that the afterdischarges by 4-AP application seem to originate from the distal axons, different from the physiological spike initiation sites of the axon initial segment or the proximal axon (Schmidt-Hieber et al., 2008). To test this notion, several experimental approaches have been carried out in this study. First, local application of 4-AP in Ca^2+^-free solution, in which indirect effects due to enhanced synaptic transmission and/or glio-transmission are supposed to be suppressed, also elicited burst firings, implicating that burst firings are triggered by the blockade of K^+^ channels solely on the distal axons. In addition, the local application of Rubi-4-AP, in combination with local illumination of blue light to the stratum lucidum in the CA3 region, reliably elicited burst firings. This finding also supported the axonal origin of the afterdischarges.

The hallmark of an ectopic spike is the occasional occurrence of spikelets, smaller amplitude all-or-none responses recorded from the soma (MacVicar and Dudek, 1981; Keros and Hablitz, 2005; Epsztein et al., 2010). Spikelets are supposed to occur by the antidromic propagation from the axon to the soma and are caused by the impedance mismatch between thin axons and the large size of the soma (Ohura and Kamiya, 2016; Michalikova et al., 2017, 2019). Intriguingly, the 4-AP-induced burst in this study was not accompanied by the smaller amplitude events. This might be due to the recordings from mossy fiber boutons, not from the soma. It was speculated that the smaller size of mossy fiber boutons than the size of granule cell soma does not cause impedance mismatch as expected for the antidromic propagation to the soma. Enriched expression of Na^+^ channels on the mossy fiber boutons (Engel and Jonas, 2005) might also explain why the spikelets were not recorded in this recording configuration. The all-or-none nature as well as the generation by focal application of 4-AP support the notion that the recorded afterdischarge reflects ectopic action potentials generated around the recording sites.

A striking finding is that a surgical cut between the dentate gyrus and the CA3 region abolished the burst firings induced by local application of 4-AP. The cut is expected to disconnect the granule cell soma physically from the distal portion of the mossy fiber axons where the recorded boutons are located. Since the connected soma was required for the generation of the axonal bursts, it was speculated that the burst firings were initially triggered by the action potentials propagated orthodromically from the soma to the distal axon. The duration of the action potentials at the distal axons was prolonged and might cause a subthreshold resonance of membrane potentials at the site of K^+^ channel inhibition, which in turn generates ectopic bursts at the distal axons and reverberates to the soma antidromically, as supported by the simulation results (Figure 5). In this scenario, spontaneous firings of the granule cells are needed to trigger the ectopic burst firings. Although the spontaneous firings of the granule cells are reported to occur less often (Henze et al., 2000), some spontaneous firings are observed during the baseline recordings (Figures 1, 2) in these experimental conditions. In support of this notion, the baseline recording after the surgical cut displayed much fewer spontaneous firings (Figure 3) than in the uncut slices in the above condition.

Another line of support of ectopic burst generation was obtained in the computer simulation approach using a model of hippocampal mossy fibers incorporating the structure of *en passant* axon and the conductances obtained by subcellular recording from the mossy fiber terminals. By removing the potassium conductance (*g*_K_) from the distal axons, which mimic the conditions of local blockade of K^+^ channels, the broadening of the action potentials and the reverberating burst firings are reproducibly induced in the model simulation. When the soma was stimulated by injecting the current pulses, action potentials faithfully propagated orthodromically to the distal axons. In turn, the subthreshold resonance of membrane potential at the site of K^+^ channel inhibition triggers repetitive firings at the surrounding boutons and propagates antidromically toward the soma, as demonstrated in the simulation.

### Impact of ectopic burst firings

Ectopic spiking from different sites of the physiological spike initiation is an important biological implication in the non-canonical mode of neuronal signaling (Avoli et al., 1998; Amir et al., 2002; Bucher and Goaillard, 2011; Coggan et al., 2015; Alpizar et al., 2019). In physiological conditions, axonal spikes are triggered from the proximal portions of the unmyelinated mossy fiber axons (Schmidt-Hieber et al., 2008; Ohura and Kamiya, 2016), as from the axon initial segments in most myelinated axons in the central nervous system. Although the ectopic spike is expected to enhance the signal flows in the neuronal circuits, a barrage of antidromic spikes during repetitive firings is expected to suppress the normal signal flow of orthodromic propagation by the influence of the refractory period or “collision” of action potentials. Regarding the net influence of the ectopic burst firings, it is also needed to take into account the well-known detonator functions of the mossy fibers input that drive postsynaptic firing through their robust frequency-dependent facilitation with an extremely wide dynamic range (Henze et al., 2000). It should be noted that the local imbalance of the sodium and potassium conductances (Coggan et al., 2010) not only affects the waveform of action potentials but also modulates the strength and/or the timing of signal flow in the neural circuits. The functional significance and consequence of the ectopic burst firings await to be clarified in future investigations.

### Distal axon as a cellular oscillator

This study demonstrated that the distal axon can be the intrinsic oscillator of the repetitive firings with the local imbalance of potassium conductance over sodium conductance (Coggan et al., 2010). As for the mechanisms underlying network oscillations, it is important to distinguish several independent processes, i.e., initial triggers, maintenance of sustained activities, synchronization of a population of neurons, and termination of the repetitive firings. Although maintenance is suggested to be attributed to the recurrent network of excitatory and inhibitory connections and synchronization is suggested to be attributed to multi-cellular coupling by gap junctions, the mechanisms underlying the trigger of oscillations mostly remain elusive. This study illuminates the distal axon as a potential site for triggering oscillation of repetitive action potentials. This notion is in line with those obtained in the previous studies showing that several models of experimental hyperexcitability were initiated ectopically from the distal axons (Stasheff and Wilson, 1990; Stasheff et al., 1993; Pinault, 1995; Sheffield et al., 2011). The ectopic repetitive firings might be caused by the axonal localization of slow Na^+^ conductance (Kapoor et al., 1997; Ohura and Kamiya, 2018b) or HCN channels (Elgueta et al., 2015; Roth and Hu, 2020; Rózsa et al., 2023). It is also indicative of the changes in the local microenvironment surrounding distal axons resulting in the ectopic burst firing (Louvel et al., 1994; Zhao et al., 2011; Deemyad et al., 2018; Thome et al., 2018).

In summary, the mechanism underlying hyperexcitability has been examined by a combination of experiments and model simulations in this study. All the results are in support of the generation of burst firings from distal axons, an ectopic site from the physiological spike initiation at the proximal axon or the axon initial segment. The functional significance of the ectopic spiking of the distal axons awaits further investigation.

## Data availability statement

The raw data supporting the conclusions of this article will be made available by the authors, without undue reservation.

## Ethics statement

The animal study was approved by the guidelines for the care and use of laboratory animals at Hokkaido University. The study was conducted in accordance with the local legislation and institutional requirements.

## Author contributions

HK: Conceptualization, Data curation, Formal analysis, Funding acquisition, Investigation, Methodology, Software, Validation, Writing – original draft, Writing – review & editing.

## Footnotes

^1^https://modeldb.science/263034

^2^https://modeldb.science/2015571
